# Predictors of switching antipsychotic medications in the treatment of schizophrenia

**DOI:** 10.1186/1471-244X-10-75

**Published:** 2010-09-28

**Authors:** Allen W Nyhuis, Douglas E Faries, Haya Ascher-Svanum, Virginia L Stauffer, Bruce J Kinon

**Affiliations:** 1 Eli Lilly and Company, Indianapolis, IN USA

## Abstract

**Background:**

To identify patient characteristics and early changes in patients' clinical status that best predict subsequent switching of antipsychotic agents in the long-term treatment of schizophrenia.

**Methods:**

This post-hoc analysis used data from a one-year randomized, open-label, multisite study of antipsychotics in the treatment of schizophrenia. The study protocol permitted switching of antipsychotics when clinically warranted after the first eight weeks. Baseline patient characteristics were assessed using standard psychiatric measures and reviews of medical records. The prediction model included baseline sociodemographics, comorbid psychiatric and non-psychiatric conditions, body weight, clinical and functional variables, as well as change scores on standard efficacy and tolerability measures during the first two weeks of treatment. Cox proportional hazards modeling was used to identify the best predictors of switching from the initially assigned antipsychotic medication.

**Results:**

About one-third of patients (29.5%, 191/648) switched antipsychotics before the end of the one-year study. There were six variables identified as the best predictors of switching: lack of antipsychotic use in the prior year, pre-existing depression, female gender, lack of substance use disorder, worsening of akathisia (as measured by the Barnes Akathisia Scale), and worsening of symptoms of depression/anxiety (subscale score on the Positive and Negative Syndrome Scale) during the first two weeks of antipsychotic therapy.

**Conclusions:**

Switching antipsychotics appears to be prevalent in the naturalistic treatment of schizophrenia and can be predicted by a small and distinct set of variables. Interestingly, worsening of anxiety and depressive symptoms and of akathisia following two weeks of treatment were among the more robust predictors of subsequent switching of antipsychotics.

## Background

Antipsychotic medications are mainstays in the clinical management of schizophrenia. Although generally effective in ameliorating core manifestations of the disease, some patients experience only suboptimal responses or are intolerant of the medication. This may include insufficient improvement or even worsening of symptoms, as well as a variety of treatment-related adverse events [1,2]. Under such clinical circumstances, a change (i.e., switch) in the antipsychotic medication regimen is warranted, representing a rational rescue treatment option in the hope that the switch will result in better treatment outcomes for the patient [3-10].

Reasons for antipsychotic switching or discontinuation are varied [2,11]; however, data from naturalistic clinical settings on the frequency of antipsychotic switching, as well as the timing and predictors of such medication changes, are limited. Previous studies evaluating predictors of switching [12,13] assessed a relatively narrow range of variables and did so for patients who may not be representative of those treated in usual outpatient care settings. Furthermore, previous research assessed predictors of medication switching at discrete time points [12,13], thus providing a time-limited context for this dynamic treatment practice. For example, the study by Weinmann and colleagues [13] evaluated switching from first-generation to second-generation antipsychotics among inpatients with schizophrenia. However, hospitalizations are often triggered by poor treatment responses or nonadherence with the previous antipsychotic regimen and thus inherently necessitate medication alterations (switches). Furthermore, inpatient data are not representative of outpatient clinical practice settings. Another study, by Sernyak and colleagues [12], used an administrative claims database to identify predictors of medication switching among patients with schizophrenia treated at the Veterans Health Administration. Independent variables included information about service utilization, sociodemographic, and a few clinical variables. The study concluded that high levels of outpatient and inpatient service use were the most powerful predictors of switching, while sociodemographic, institutional, diagnostic, and functional measures were also predictive in some cases.

The purpose of our study was to expand current research and identify individual patient characteristics that best predict switching of antipsychotic medications among predominately outpatients treated for schizophrenia and related disorders. This study is focused on patients who switch antipsychotic medication (switchers), the ones who constitute the pool of patients who remain engaged in treatment, for whom the clinicians have to consider different treatment choices to replace the current therapy. Unlike patients who drop out of treatment (discontinuers), the switchers show interest in further treatment and are available for initiation of alternative treatment options. Our previous research [14] has suggested that although treatment discontinuation for any cause (switch or discontinuation) is an important proxy measure of a medication's effectiveness, the differences between antipsychotic medications on this proxy measure may be primarily driven by switching of the medication (when switching is a study option) rather than discontinuation. Our prior research [15] has also helped to show that in the treatment of patients with schizophrenia, switching antipsychotics may be a meaningful marker of treatment failure, considering its significant association with more frequent and more rapid use of acute care services (hospitalization and crisis services) compared with persons remaining on their initial treatment.

Therefore, to identify individual patient characteristics that best predict switching of antipsychotic medications in the treatment of schizophrenia, we conducted a *post-hoc *analysis of a one-year randomized, open-label, multisite cost-effectiveness study of antipsychotic medications in the treatment of schizophrenia in the United States. Consistent with the parent study protocol, switching of the initially randomized antipsychotic was permitted if clinically warranted [16-18]. The objectives of the current study were to assess the frequency of antipsychotic switching, the time to switching, and the patient and treatment characteristics that best predict subsequent switching of antipsychotics over a one-year period. We used numerous independent variables to reflect baseline patient sociodemographic and clinical characteristics, as well as early clinical changes observed within the first two weeks of antipsychotic therapy.

## Methods

### Data source

We used data from a Lilly-sponsored, randomized, open-label, one-year, multicenter, cost-effectiveness study of antipsychotics in the treatment of schizophrenia (HGGD). This study compared the cost-effectiveness of initial treatment with olanzapine versus a "fail-first" on typical antipsychotics (then olanzapine if indicated) and versus initial treatment with risperidone. The study was conducted at 21 sites in 15 states from May 1998 through September 2002, and its primary findings have been published [17]. Briefly, the study found that requiring failure on less expensive antipsychotics before use of olanzapine did not result in total cost savings, despite significantly higher antipsychotic costs with olanzapine.

The study included patients who were deemed by their physicians to warrant a change in their antipsychotic medication, using broad eligibility criteria: patients aged 18 years or older with a DSM-IV diagnosis of schizophrenia, schizoaffective, or schizophreniform disorder, provided they scored ≥18 on the Brief Psychiatric Rating Scale (BPRS) [19]. No patient was excluded because of comorbid substance use disorders or other psychiatric or medical comorbidities, unless the condition was severe. Almost all enrollees were outpatients (95%).

At study initiation, patients were randomized to one of three open-label treatment groups: olanzapine (n = 229); risperidone (n = 221); or first-generation antipsychotic of physician's choice (n = 214). Patients remained on their initially assigned medication for at least eight weeks, after which, if clinically warranted per clinicians' judgment, patients' regimens could be changed to a different antipsychotic agent. Patients were assessed at baseline and at five predetermined post-baseline visits (2 weeks; 2, 5, 8, and 12 months post-baseline), regardless of the time of medication switch. The protocol and consent procedures were approved by institutional review boards, and after being provided with a complete description of the study, signed consent forms were obtained from patients prior to participation.

### Assessments and predictor variables

A wide range of independent variables was evaluated in patients who switched antipsychotic treatment compared with their counterparts who completed the study without a switch.

Baseline sociodemographic variables included age, gender, race/ethnicity, educational attainment, marital status, employment, and insurance status. Baseline clinical variables included symptom severity, quality of life, functional status, safety and tolerability, hospitalizations and emergency services in the year prior to enrollment, illness duration, use of antipsychotic and switching of antipsychotics in the prior year, prior adherence with antipsychotics defined as the medication possession ratio (MPR, the proportion of days with any antipsychotic during the one-year prior to enrollment), pre-existing comorbid psychiatric and non-psychiatric conditions (assessed at enrollment, including depression and insomnia), total number of pre-existing comorbid conditions of any type, past incarcerations, and past suicide attempts.

To help identify predictor variables that emerge during the early phase of treatment ("early on-treatment variables"), a wide range of variables was measured at baseline and again at two weeks post-baseline to compute a two-week change score. These "on-treatment variables" reflected measures of symptomatology, quality of life, functional status, safety, and tolerability. Changes occurring during the first two weeks of treatment were used based on previous research showing that most improvements are observed during the first two weeks of treatment [20] and that early non-response to medication is a robust predictor of subsequent non-response to the same antipsychotic medication [21-24].

Symptomatology was assessed using the Positive and Negative Syndrome Scale (PANSS) [25] total score and the five PANSS factor subscales [26]. Quality of life was assessed using the 17 subscales (nine subjective, eight objective) of the Lehman Quality of Life Interview [27]. Functional status was measured with the eight subscale scores and two composite scores of the MOS 36-item short form health survey **(**SF-36) [28]. Global assessment of functioning (GAF) was also included [29].

Safety and tolerability (at baseline and again following two weeks of treatment) was determined using clinician-rated scales for akathisia [30] and extrapyramidal symptoms [31]. Baseline body weight and treatment-emergent weight gain during the first two weeks of treatment were assessed. The study did not include measures of metabolic parameters (other than weight) or prolactin levels.

### Statistical analysis

Data from patients who discontinued their initially assigned antipsychotic and were switched to another antipsychotic within 14 days of medication discontinuation (switchers) were compared with those who completed the one-year study on their randomized medication (nonswitchers). Patients who discontinued the study early (dropouts) without a switch prior to study discontinuation were not included in the present analysis. The switcher group was aggregated across the three medication treatment groups, as was the nonswitcher group. This was done since the assessed reasons for switching (i.e., patient request, lack of efficacy, medication intolerability, other) did not significantly differ among the three treatment groups, although the switching rate was significantly lower for patients randomized to olanzapine (14%) compared to a typical antipsychotic (53%, p < .001) and to risperidone (31%, p < .001) [17].

Chi-square, Fisher's exact, Wilcoxon rank-sum, and independent *t *tests were used to conduct univariate comparisons of all potential predictor variables between switchers and nonswitchers. The relationship between each potential predictor variable and time to switching was assessed univariately using Cox proportional hazards regression. Time to switching was defined as remaining in the study on the initially assigned medication without switching. If a patient's regimen was not switched over the one-year study period, the survival time (time to switching) was censored either at study completion or when the patient prematurely discontinued the study.

Predictor variables identified from the above analyses (with p < .05 from either the univariate survival analysis or the comparisons between switcher and nonswitcher groups) were used as initial variables in fitting a predictive model using Cox proportional hazards, with the outcome variable being time-to-switch. Using this initial model as a starting point, the final predictor variables were determined by utilizing a manual stepwise procedure (with p < .05 as the criterion for variables to either enter or stay in the model), using all of the potential predictor variables (including variables not in the initial model). Once the final predictors were determined, all two-way interactions involving the final predictor variables were tested for inclusion in the final model. Significance was defined a priori at a two-tailed alpha ≤.05.

As a sensitivity analysis, the final predictive model was refit on only the set of patients who did not continue on the same treatment at randomization as they were already taking pre-baseline. This was done to address the possibility that continuing on the same treatment might be predictive of earlier or later switching.

To illustrate the associations between each of the final predictor variables and time to switching, a graph of the Kaplan-Meier estimated survival distribution was produced in a univariate fashion separately for each predictor variable.

## Results

**Figure 1:**
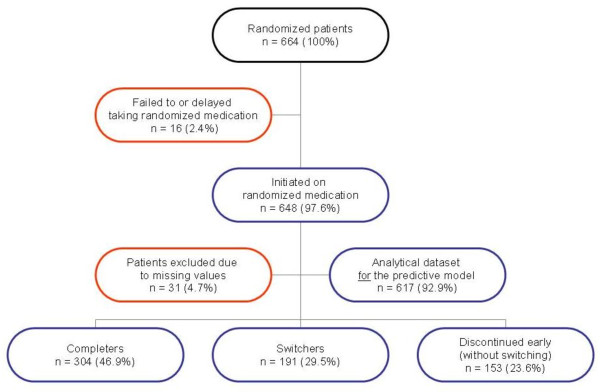
**Analytical Sample**.

Of 664 patients enrolled in the parent study, 16 (2.4%) either failed to or delayed taking their randomized medication, leaving an analysis dataset of 648 patients (Figure 1). A total of 304 (46.9%) of the 648 patients completed the one-year study on the randomly assigned medication (i.e., nonswitchers), whereas 191 (29.5%) switched to a different antipsychotic (i.e., switchers). The remaining 153 patients (23.6%) discontinued participation in the study without switching to a new medication prior to dropout. These "early discontinuers" had a mean age of 41.3, with 70% of them Male, 49% Caucasian, 53% Single/Never Married, and their mean Total PANSS score was 88.3. Only 14% of this group were Employed, but 55% had a Substance Abuse diagnosis in the past year, and 60% of them have been Incarcerated. Among medication switchers, the reasons for the switching were noted as patient decision (34.6%), lack of medication efficacy (27.7%), adverse event (22.5%), and other or unknown reasons (15.2%). Results of the univariate analyses revealed several variables that were significantly (p < .05) associated with switching: female gender; no previous antipsychotic treatment in the year before study initiation; no current or lifetime diagnosis of substance use disorder; pre-existing insomnia; and early (within two weeks post-baseline) worsening of depressive symptoms per scores on the depression/anxiety subscale of the PANSS (Table 1). Baseline body weight and change in weight during the first two weeks of treatment did not predict switching in this study. Similarly, quality of life, functional variables, employment, insurance status, and adherence level in prior year (per MPR) were not predictive of switching or earlier switching.

**Table 1:** Baseline Characteristics and Selected Univariate Predictors of Switching

Variable	All patients (N = 648)	Switchers (n = 191)	Completers (n = 304)	p value (switchers vs. completers)^1^	p value (univariate survival comparison)^2^
Age, mean (SD), y	42.9 (12.1)	42.8 (12.5)	43.7 (11.8)	0.404	0.724

Female, n (%)	239 (37%)	87 (46%)	106 (35%)	0.018	0.013

Caucasian, n (%)	352 (54%)	107 (56%)	170 (56%)	0.998	0.890

Currently employed, n (%)	122 (19%)	36 (19%)	64 (21%)	0.568	0.903

Illness duration, mean (SD), y	20.6 (12.2)	20.6 (12.6)	21.3 (12.1)	0.577	0.981

Hospitalized, previous year, mo mean (SD)	0.51 (1.53)	0.40 (1.25)	0.49 (1.54)	0.450	0.343

Switch in previous year, n (%)	85 (13%)	23 (13%)	44 (15%)	0.588	0.650

Any antipsychotic previous year, n (%)	579 (89%)	165 (86%)	284 (93%)	0.011	0.095

Substance abuse diagnosis, n (%)	289 (45%)	70 (37%)	135 (44%)	0.111	0.038

Schizoaffective diagnosis, n (%)	280 (43%)	80 (42%)	130 (43%)	0.852	0.795

Ever attempted suicide, n (%)	235 (38%)	78 (43%)	99 (34%)	0.064	0.084

Ever incarcerated, n (%)	284 (46%)	71 (38%)	127 (43%)	0.295	0.075

PANSS total score, mean (SD)	86.8 (20.0)	84.5 (18.8)	87.4 (21.1)	0.120	0.102

PANSS Davis, positive symptoms, mean (SD)	22.3 (6.3)	21.9 (5.8)	22.1 (6.6)	0.796	0.545

PANSS Davis, negative symptoms, mean (SD)	21.3 (7.0)	20.4 (6.8)	21.9 (7.3)	0.026	0.043

PANSS Davis, impulsivity/hostility, mean (SD)	8.9 (3.6)	8.9 (3.9)	8.9 (3.6)	0.990	0.675

PANSS Davis, disorganized thought, mean (SD)	21.2 (6.0)	20.7 (5.6)	21.6 (6.3)	0.096	0.085

PANSS Davis, anxiety/depression, mean (SD)	13.0 (4.2)	12.7 (4.0)	13.0 (4.3)	0.411	0.445

SF-36 Physical component score, mean (SD)	-0.43 (1.04)	-0.43 (1.05)	-0.42 (1.01)	0.963	0.803

SF-36 Mental component score, mean (SD)	-1.08 (1.33)	-1.14 (1.33)	-0.82 (1.28)	0.009	0.106

Barnes Akathisia	0.24	0.20 (0.53)	0.25 (0.62)	0.356	0.273

item #1, objective, mean (SD)	(0.57)				

Barnes Akathisia, total score, mean (SD)	0.99 (1.58)	0.96 (1.46)	0.95 (1.67)	0.954	0.813

GAF functioning, current score, mean (SD)	46.1 (12.9)	47.2 (13.4)	47.0 (13.2)	0.842	0.323

Antidepressant Drugs taken (%)	297 (46%)	85 (45%)	212 (46%)	0.667	0.340

Anti-Anxiety Drugs taken (%)	187 (29%)	54 (28%)	133 (29%)	0.850	0.810

Antiparkinsonian Drugs taken (%)	315 (49%)	93 (49%)	222 (49%)	1.000	0.453

Patient weight, mean (SD), kg	86.7 (20.7)	86.9 (21.2)	87.7 (20.4)	0.706	0.947

Body mass index, mean (SD)	29.7 (6.8)	30.4 (7.1)	29.8 (6.9)	0.426	0.229

Patient weight change from baseline to 2 weeks, mean (SD), kg	+0.8 (2.8)	+0.7 (3.2)	+0.8 (2.4)	0.706	0.646

Pre-existing depression, n (%)	96 (15%)	36 (19%)	39 (13%)	0.073	0.056

Pre-existing insomnia, n (%)	66 (10%)	26 (14%)	24 (8%)	0.047	0.030

PANSS Davis anxiety/depression, change from baseline to 2 weeks, mean (SD)	-1.42 (3.47)	-1.19 (3.74)	-1.88 (3.36)	0.041	0.093

Barnes Akathisia objective score, change from baseline to 2 weeks, mean (SD)	-0.04 (0.59)	+0.02 (0.62)	-0.05 (0.57)	0.193	0.058

Abbreviations: PANSS, positive and negative syndrome scale; SD, standard deviation; SF-36, Medical Outcomes Study 36-item short form health survey; GAF, global assessment of functioning scale.

**^1^**Univariate descriptive statistic comparisons, using unpaired t-tests for numeric data (confirming with Wilcoxon rank-sum test, if non-normality was suspected) or chi-square tests (or Fisher's exact test, for small numbers) for categorical data.

^2^Univariate survival comparisons, using Cox proportional hazards models with only the one single variable.

According to the multivariate regression model, six variables were found to significantly predict (p < .05) antipsychotic switching: four baseline patient characteristics and two early treatment variables (Table 2). The four baseline characteristics were female gender, pre-existing depression, lack of antipsychotic medication use in the year prior to the study, and lack of substance use disorder. The two early treatment variables were worsening of anxiety/depression symptoms (per PANSS subscale score) and worsening of akathisia (per Barnes Akathisia objective score) in the first two weeks of treatment. According to the hazard ratios, women were 37.6% more likely to switch earlier than their male counterparts, and patients with pre-existing depression were 48.4% more likely to switch before similar patients without pre-existing depression. Alternatively, participants less likely to switch medications included those who were treated with any antipsychotic in the year before the study (38.3% less likely) and those diagnosed with substance use disorder (26.9% less likely).

**Table 2:** Proportional Hazards Model of Predictor Variables

Variable	Cox proportional hazards model parameter	p value	Hazard ratio (95% confidence interval)
Female	+0.3192	0.0335	1.376 (1.025-1.847)

Any antipsychotic in the previous year	-0.4836	0.0262	0.617 (0.403-0.944)

Substance abuse diagnosis	-0.3133	0.0457	0.731 (0.538-0.994)

Pre-existing depression condition	+0.3948	0.0344	1.484 (1.029-2.139)

PANSS Davis anxiety/depression, change from baseline to 2 weeks	+0.0498 (per 1-point increase)	0.0320	1.051 (1.004-1.100) (per 1-point increase)

Barnes akathisia objective score, change from baseline to 2 weeks	+0.2962 (per 1-point increase)	0.0398	1.345 (1.014-1.783) (per 1-point increase)

Abbreviations: PANSS, positive and negative syndrome scale.

There were two early treatment variables significantly predictive of an increased likelihood of earlier switching, one associated with medication efficacy and the other with medication intolerability. These variables were an increase (worsening) of the PANSS depression/anxiety subscale score and an increase (worsening) of the Barnes Akathisia objective score (Table 2) in the first two weeks of treatment. According to the hazard ratios, for every 1-point increase on the PANSS depression/anxiety subscale score, patients had a 5.1% higher likelihood of switching sooner than those without such changes in scores. A 2-point increase in the PANSS depression/anxiety subscale score was associated with a 10.5% higher likelihood of switching earlier, whereas a 1-point decrease (improvement) reduced the likelihood of an earlier switch by 4.9%. Furthermore, for every 1-point increase on the Barnes Akathisia objective score, there was a 34.5% increased likelihood of switching earlier, whereas each 1-point drop was associated with a 25.7% decreased likelihood of earlier switching as compared with patients whose Barnes Akathisia scores did not drop.

In order to assess how much each predictor has contributed to the model, we determined how much the likelihood ratio changed when each of the six predictor variables was dropped from the model. This provided the rank order from the most to the least significant predictor (smaller number is better, as it indicates a greater effect of dropping that predictor): worsening of PANSS anxiety/depression score during the first two weeks of treatment (likelihood ratio = 19.325); female gender (likelihood ratio = 19.364); lack of antipsychotic medication use in the prior year (likelihood ratio = 19.429); worsening of akathisia in the first two weeks of treatment (likelihood ratio = 19.507); pre-existing depression (likelihood ratio = 19.714); and lack of substance use disorder (likelihood ratio = 20.149). Although Cox proportional hazards regression does not provide a simple statistic (like R-square) to measure the percentage of the total variance in switching explained by the model, the relative "fit" of the model, as assessed by comparing the model with versus without the six predictor variables, indicated a highly significant fit (likelihood ratio = 23.836, p = .0006).

**Figure 2:**
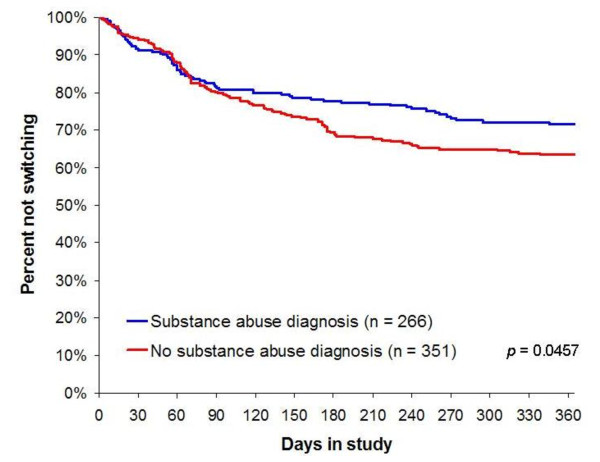
**Current or Previous Substance Abuse Diagnosis**.

**Figure 3:**
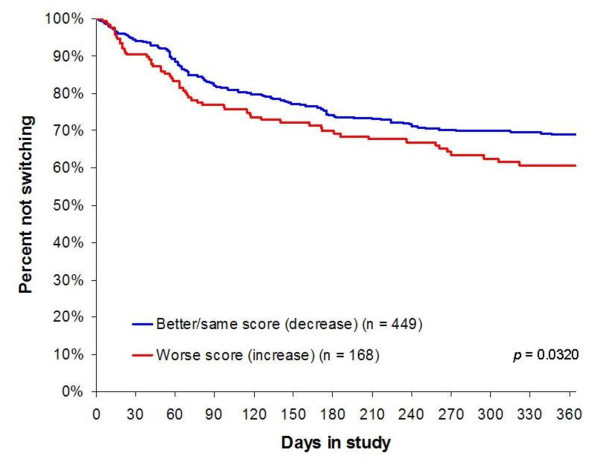
**PANSS Davis Anxiety/Depression Change From Baseline to 2 Weeks**.

**Figure 4:**
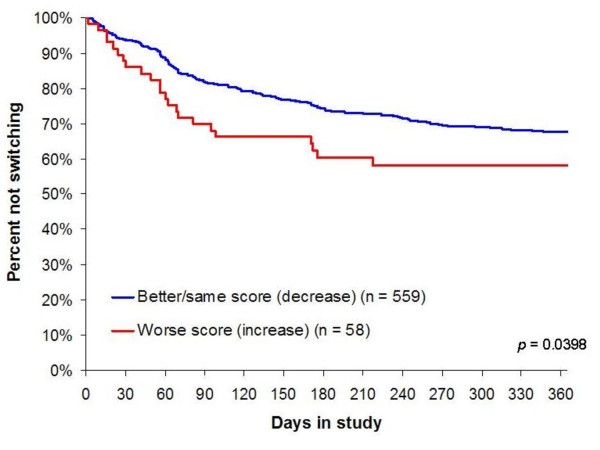
**Barnes Akathisia Objective Score Change From Baseline to 2 Weeks**.

In the survival plots in Figures 2, 3, 4, three of the six significant (p < .05) predictors of switching (or earlier switching) are illustrated. The figures augment information about the likelihood of switching with information about the time to switching over the one-year study. Of note, worsening in medication efficacy and tolerability within the first two weeks of treatment is clearly significantly associated with earlier switching (Figures 3 and 4).

## Discussion

In this *post-hoc *analysis of a randomized, open-label study conducted in naturalistic, predominately outpatient settings, nearly one in three (29%) patients switched before completing one year of therapy with the initially assigned antipsychotic medication. Switching antipsychotics was best predicted by six variables: four baseline and two early on-treatment variables. These included, from most to least statistically significant predictor: worsening of PANSS anxiety/depression score during the first two weeks of treatment, female gender, lack of antipsychotic medication use in the prior year, worsening of akathisia in the first two weeks of treatment, pre-existing depression, and lack of substance use disorder. These six variables were significantly predictive of both switching and of an earlier time to switch. To our knowledge, this is the first study to document patient-level risk factors for earlier switching.

Current findings might help inform clinical decision-making in usual practice. Effectively tailoring treatment regimens to patients and optimizing early treatment responses are pivotal challenges in psychiatry. For at least four decades, researchers have sought predictors of treatment outcomes after prescribing antipsychotic medications, with a focus on baseline patient variables (i.e., "moderators") and on-treatment variables (i.e., "mediators") [32]. Findings that early worsening in depressive and anxiety symptoms and in akathisia during the first two weeks of treatment predicted switching or earlier switching support the need for early monitoring of antipsychotic efficacy, tolerability, and safety to optimize treatment outcomes.

Given the observed associations, medication switching (as well as early medication discontinuation for any cause) may constitute a proxy or surrogate marker of treatment failure in many patients. This is important because treatment failure often translates into relapse, which is one of the costliest aspects of schizophrenia management in both economic and human terms [6,33-35]. The sooner such an adverse outcome can be predicted, the sooner treatment can be modified to help avert it.

In addition to early-treatment predictors (mediators), a number of baseline patient characteristics (moderators) significantly predicted switching of medication and earlier switching. Patients who did not use antipsychotic medications in the year preceding the study were more likely to switch or require an earlier switch, likely reflecting prior nonadherence with antipsychotic medications in these chronically and moderately ill patients. Previous research by our group demonstrated that patients with schizophrenia who were enrolled in a large three-year prospective observational, noninterventional study (US-SCAP) and were nonadherent to antipsychotic medication regimens in the six months before enrollment were over four times more likely to subsequently discontinue such treatment for any cause [36].

In the present study, women with schizophrenia were also significantly more likely than their male counterparts to switch medications or evidence an earlier medication switch. This finding, however, may reflect ascertainment bias, in that women with schizophrenia may, in general, use mental health services more frequently than their male counterparts [37]. Increased service use (e.g., physician visits) might in turn be associated with a higher likelihood of detecting a suboptimal treatment response or a treatment-emergent adverse event culminating in medication switching [38].

We also found that patients diagnosed with a substance use disorder were less likely to switch antipsychotic medications and less likely to switch earlier. This predictor seemed, at first, somewhat at odds with previous research, especially with findings of a large, prospective, observational study in which patients with schizophrenia with concurrent substance abuse problems were more likely to discontinue antipsychotic regimens for any cause [14]. However, all-cause medication discontinuation is composed of medication switching and study discontinuation, two components on which patient subgroups seem to differ. The importance of separating switchers from study discontinuers was illustrated in a previous analysis of the current study dataset (HGGD). In that *post-hoc *analysis [14], patients with substance use were significantly more likely to discontinue their medication and to withdraw from the study rather than switch medications. Furthermore, the finding that patients with substance use disorders were less likely to switch antipsychotic medications and less likely to switch earlier might also represent the confounding by gender, because switchers were more likely to be women and substance use is less prevalent among women than men [37].

Arguably one of the most important findings of the current study is that affective symptoms and, specifically, depressive and anxiety symptoms (pre-existing depression and worsening of depression and anxiety symptoms during the first two weeks of treatment), appear to be robust predictors of subsequent switching or earlier switching of medication. Current findings are consistent with previous research demonstrating that depressive symptoms are associated with a significantly higher propensity to discontinue treatment for any cause [39-41]. The study by Kinon and colleagues [40] investigated this aspect in some detail, with a *post-hoc *analysis of pooled data from four antipsychotic trials for the treatment of schizophrenia (n = 1,627). That study showed that patients with a 4-point improvement in PANSS depression/anxiety subscore were significantly less likely to discontinue treatment, and an early response in depressive/anxiety symptoms was associated with a 50% greater likelihood of study completion. These, along with the current findings, emphasize the prognostic value of affective symptoms, especially depression and anxiety, in the treatment of patients with schizophrenia.

The current findings also highlight the importance of early worsening akathisia as a predictor of medication switching. These results are consistent with prior research showing that akathisia is bothersome and distressing to patients [42-44] and is associated with medication nonadherence [45,46].

It is of interest that no association was found between medication switching and baseline body weight, BMI, or treatment-emergent body weight in the first two weeks of treatment. It is possible that health concerns about treatment-emergent weight gain were not yet pronounced during the study period (through 2002), thus did not lead to medication switching by the clinicians. It is also possible that clinicians have recognized the association between therapeutic response and greater treatment-emergent weight gain [47-50] and opted, after risk-to-benefit assessment, not to switch most of these patients' medications. These hypotheses are speculative, as further research is needed to help clarify reasons for medication continuation and reasons for medication discontinuation from the patients' and clinicians' perspectives.

The CATIE schizophrenia study [2,4,6,7] found that individuals who had "continuation" (randomized to the same antipsychotic they had received prior to study entry) had significantly longer times to all-cause discontinuation. Indeed, when this variable was tested in our study, it was a significant predictor (p < .001) of switching. When, as a sensitivity analysis, the final predictive model was re-fit to only the set of patients (n = 442) who did not have continuation, the results showed hazard ratios which were directionally consistent with the original predictive model.

Study findings need to be evaluated in light of its limitations. First is the study's *post-hoc *nature, suggesting the need for additional longitudinal research to confirm the findings in an a priori manner. Second, patients enrolled in this study were primarily chronically ill outpatients with schizophrenia with about 20 years of illness duration, who agreed to participate in a randomized study; therefore, our findings may not be applicable to first-episode patients to inpatients, or to patients treated in a usual care setting. In addition, this study was conducted during a timeframe when second-generation antipsychotics were fairly new to the market, so it is not clear how changes in the standards of treatment over time may have impacted the switching decision-making process. Another limitation is the time-to-event survival analysis: whether "censored" (discontinued from the study) subjects would have soon switched medication cannot be determined since they were not followed up after dropping out of the study. This limitation may help explain the finding that a lack of substance use disorder was predictive of switching, because substance users are prone to study discontinuation rather than to switch medications [14,51]. Traditional survival analyses assume that censoring is independent of the outcome event (in this case, switching), an assumption that is not likely to be fully satisfied. Next, although a relatively wide range of potential predictors of switching was examined, the list was not exhaustive. The study lacked data on changes in metabolic parameters (besides body weight) and prolactin levels, and these changes may lead some clinicians to switch medications. Consequently, further research is needed to incorporate such important safety measures when assessing predictors of switching. In addition, the most frequent reason for switching was "patient's decision," thus limiting the ability to discern what may have triggered the switch for a substantial proportion of the patients. Finally, but most importantly, this study focused on switchers and not on patients who discontinued the study early, although information about discontinuers is also of interest and clinical importance. Therefore, further research is needed to compare baseline and early treatment characteristics of switchers and discontinuers and assess whether predictors of switching differ from predictors of medication discontinuation in the treatment of patients with schizophrenia. Despite its limitations, this study has a number of strengths. In addition to conducting survival analyses to assess time to switching, the study used liberal eligibility criteria and was conducted in naturalistic settings, which may enhance the ability to generalize the current findings to the wider U.S. outpatient schizophrenia patient population. Another strength of the present study is the broad spectrum of patient-level variables examined as potential predictors and the use of "early on-treatment" variables to assess the predictive value of early changes in patients' status to reflect the medication's early efficacy, tolerability, and safety.

## Conclusions

In conclusion, switching antipsychotic medications is common in the outpatient management of schizophrenia and can be considered a surrogate for treatment failure in many patients. Early suboptimal treatment outcomes in terms of efficacy (worsening of depressive/anxiety symptoms) and tolerability (worsening of akathisia) significantly predict switching or an earlier time to switch. Patient characteristics predictive of switching earlier included female gender, a history of depression, and the lack of recent use of antipsychotics. Further longitudinal studies are needed to evaluate and replicate these findings.

## Competing interests

The authors are full-time employees of and minor shareholders of Eli Lilly and Company.

## Authors' contributions

All authors participated in the study conduct and design. AWN, DEF, and HA-S provided oversight of the study design. AWN was responsible for the acquisition of the data. All authors participated in the interpretation of the data. AWN and HA-S prepared the manuscript with editorial assistance from Rete Biomedical Communications Corp. and revisions by all authors. All authors read and approved the final manuscript.

## Pre-publication history

The pre-publication history for this paper can be accessed here:

http://www.biomedcentral.com/1471-244X/10/75/prepub
